# The Effects of Heat Advection on UK Weather and Climate Observations in the Vicinity of Small Urbanized Areas

**DOI:** 10.1007/s10546-017-0263-0

**Published:** 2017-06-12

**Authors:** Richard Bassett, Xiaoming Cai, Lee Chapman, Clare Heaviside, John E. Thornes

**Affiliations:** 1 School of Geography, Earth and Environmental Sciences, University of Birmingham, Edgbaston, Birmingham, B15 2TT UK; 2 Chemical and Environmental Effects Department, CRCE, Public Health England, Harwell, UK

**Keywords:** Temperature observations, Urban heat advection, Urban heat island, Urbanization

## Abstract

Weather and climate networks traditionally follow rigorous siting guidelines, with individual stations located away from frost hollows, trees or urban areas. However, the diverse nature of the UK landscape suggests that the feasibility of siting stations that are truly representative of regional climate and free from distorting local effects is increasingly difficult. Whilst the urban heat island is a well-studied phenomenon and usually accounted for, the effect of warm urban air advected downwind is rarely considered, particularly at rural stations adjacent to urban areas. Until recently, urban heat advection (UHA) was viewed as an urban boundary-layer process through the formation of an urban plume that rises above the surface as it is advected. However, these dynamic UHA effects are shown to also have an impact on surface observations. Results show a significant difference in temperatures anomalies ($p\,< \,0.001$) between observations taken downwind of urban and rural areas. For example, urban heat advection from small urbanized areas ($\sim$1$\,\hbox {km}^{2}$) under low cloud cover and wind speeds of 2–3$\,\hbox {m}\,\hbox {s}^{-1}$ is found to increase mean nocturnal air temperatures by 0.6$\,^{\circ }\hbox {C}$ at a horizontal distance of 0.5 km. Fundamentally, these UHA results highlight the importance of careful interpretation of long-term temperature data taken near small urban areas.

**Electronic supplementary material:**

The online version of this article (doi:10.1007/s10546-017-0263-0) contains supplementary material, which is available to authorized users.

## Introduction

### Background

Average global air temperatures increased by 0.9$\,^{\circ }\hbox {C}$ between 1880 and 2012 (IPCC 2014). Concurrently, rapid urbanization continues to take place with 54% of the world’s population now residing in urban areas (higher in developed countries, e.g. 82% in the UK; United Nations 2014). Attempts to quantify the contribution of increasing urbanization to the rise in global temperatures show that the effect is typically an order of magnitude less than overall warming due to increasing greenhouse gas concentrations (e.g. 0.05$\,^{\circ }\hbox {C}\;\hbox {century}^{-1}$, Jones et al. 1990; 0.27$\,^{\circ }\hbox {C}\;\hbox {century}^{-1}$, Kalnay and Cai 2003; 0.05$\,^{\circ }\hbox {C}\;\hbox {century}^{-1}$, Zhou et al. 2004). However, although urban modifications to the global surface albedo have contributed relatively little to observed global warming (Pielke et al. 2002), the urban impacts on temperature that manifest at the local scale and mesoscale can be significant.

Urban fabric alters the local surface energy balance, resulting in heat storage during the day, and heat release at night. The resulting nocturnal warming, known as the urban heat island (UHI), can be locally several times larger than the observed global warming over the last century. For example, in the UK, UHI magnitudes (the maximum low-level temperature difference between the urban and rural areas) up to 9$$\,^{\circ }\hbox {C}$$ have been found in London (Kolokotroni and Giridharan 2008) and 10$$\,^{\circ }\hbox {C}$$ in Manchester (Smith et al. 2011). The magnitude of the UHI for a given urban area is approximately related to city size (Oke 1973) and varies with atmospheric stability (Azevedo et al. 2016). Indeed, even small urban areas (populations $$10^{3}$$–$$10^{4})$$, can still generate UHI magnitudes in excess of 1$$\,^{\circ }\hbox {C}$$ (Oke 1973; Ivajňsič et al. 2014). However, absolute maximum air-temperature variations are not always captured due to the challenges associated with locating stations within the urban environment (Chapman et al. 2015; Stewart 2011). Furthermore, UHI effects are not confined to temperature. The UHI has been shown to modify low-level flow (Bornstein and Johnson 1977) and increase precipitation downwind (Ackerman et al. 1978). The UHI can also have a specific impact on public health and infrastructure. For example, Heaviside et al. (2016) attributed approximately 50% of the heat-related mortality in the West Midlands, UK during the August 2003 European heatwave to the UHI.

Official weather and climate networks follow rigorous siting guidelines of the World Meteorological Organization (WMO 2008) so as to avoid undue local effects on observations (i.e. locating stations away from frost hollows, trees or urban areas). Using the classification developed by the WMO to assess the exposure of surface observations, the most representative stations are defined as those located more than 0.1 km from any anthropogenic heat sources (WMO 2008). However, Oke (2006) suggested that the actual source area affecting screen-level observations may extend up to 0.5 km. Whilst there remains no consensus in the literature with respect to source areas, indeed Parker (2006) disputed whether urban influences are present in the data at all, the heterogeneous nature of the UK landscape suggests that the search for representative stations away from the influence of urbanization is becoming increasingly difficult. However, despite these uncertainties, the majority of UHI studies only consider immediate station characteristics (up to 0.1 km, WMO 2008) and not always more distant source areas. As such, this paper sets out a methodology for exploring the role of urban heat advection (UHA) in contaminating the climate signal at weather stations located in proximity to urban areas.

### Urban Heat Advection

**Fig. 1:**
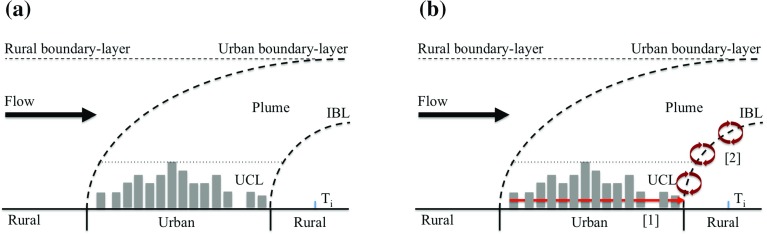
**a** Urban boundary-layer structure and urban plume (after Oke 1976). **b** Adapted urban plume to show UHA processes: *1* horizontally through the UCL, and *2* horizontal and vertical heat mixing from the urban plume shown through hypothetical eddies

Although UHI effects are generally well understood (see Arnfield 2003; Stewart 2011 for extensive reviews), the process of urban heat advection is rarely considered. Urban heat advection is the horizontal transport of heat originating from urban areas, and was conceptually considered by Lowry (1977) regarding rural weather stations contaminated by warm urban air known as an “urban environ”. To understand UHA processes, the UHI can be categorized by its vertical extent into an “urban canopy layer (UCL) UHI” and “urban boundary-layer (UBL) UHI” (Oke 1976). The UCL and UBL structure is illustrated in Fig. 1a, adapted from Oke (1976). However, this conceptual model implies physical boundaries between different layers, whereas changes across the boundaries are likely to occur gradually through turbulent mixing. Within the urban canopy-layer UHI (from ground to roof level) airflow effectively advects heat horizontally through street-canyon networks (Fig. 1b), as demonstrated by Belcher et al. (2015) using a street-network model. In contrast, the urban boundary-layer UHI (above roof level) is heated from the air below, and is affected by local to mesoscale processes. Warm, buoyant urban air forms a thermal dome that is advected horizontally if airflow is present (Fig. 1a). UBL observations have shown elevated urban thermal plumes to typically extend 10–15 km downwind of an urban area (Dirks 1974; Wong and Dirks 1978). A step change in surface properties, from urban to rural, modifies the lower part of the urban plume as heat flux and temperature profiles adapt to the rural conditions, via the internal boundary layer (IBL). The near-surface air within the IBL in the downwind proximity to the city or town is in equilibrium with the dynamical and thermal forcings at the following interfaces: (i) the local rural surface (predominately via vertical turbulent mixing), (ii) the urban plume above the IBL (predominately via vertical turbulent mixing), and (iii) the leading edge of the IBL at the downwind edge of the urban area (predominately via horizontal advection). These processes are illustrated in Fig. 1b, noting that the effects of upwind urban land use are not limited to temperature. Significant relationships have been shown to exist between upwind urban land use and turbulent heat fluxes (Rooney 2001; Rooney et al. 2005; Barlow et al. 2008).

Whilst spatial UHI studies exist and some acknowledge the urban heat island (e.g. Klysik and Fortuniak 1999; Bohnenstengel et al. 2011; Azevedo et al. 2016), they do not explicitly separate urban heat advection from the UHI signal (i.e. (i) the locally generated UHI component with intensity dependent upon underlying land use, and (ii) the urban heat advection that is generated from upwind urban land use). To decompose these components a methodology was developed by Heaviside et al. (2015) that used a time-mean 2-m air-temperature field created from mesoscale modelling to deduce urban heat advection. This methodology was subsequently adapted for use with observations from a high-density urban observation network (Birmingham Urban Climate Laboratory, Warren et al. 2016) where a significant UHA signal was found and linked to the upwind normalized difference vegetation index, a proxy for urban fraction, at city scale (Bassett et al. 2016). Whilst this methodology is able to demonstrate urban heat advection, there remain several identifiable research gaps: (i) UHA effects on global temperature series are rarely considered, (ii) the extent of urban heat advection arising from small urban areas is unknown, and (iii) station metadata do not include source areas, i.e. proximity of rural stations to urban areas. Therefore, the aim of our study is to develop previous UHA methodologies (Brandsma et al. 2003; Heaviside et al. 2015; Bassett et al. 2016) in order to quantify the influence of urban heat advection on the UK Met Office weather and climate observation network from small urban areas.

## Methods and Background

### Building Fraction Data

**Fig. 2:**
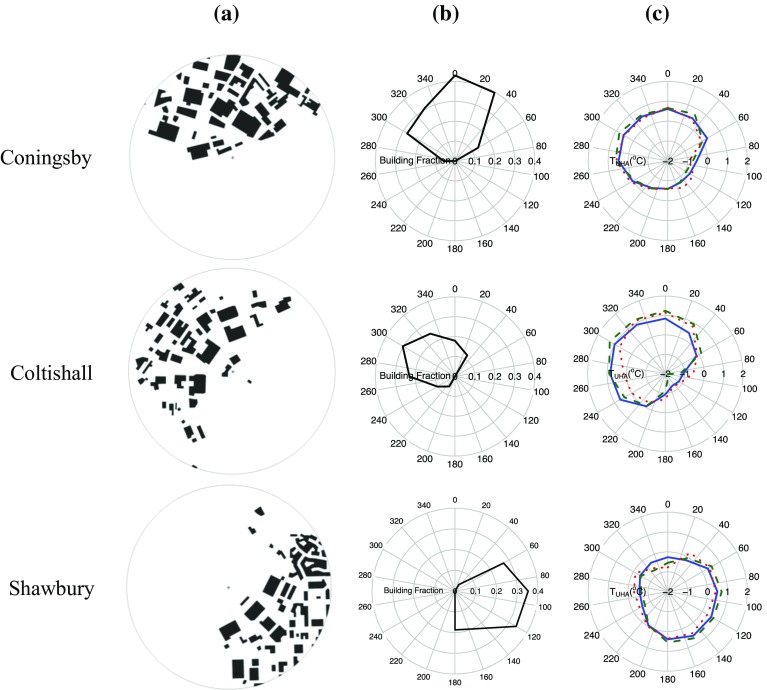
**a** Ordnance Survey VectorMap building data in a 0.5-km radius around the Met Office station at Coningsby, Coltishall and Shawbury (*centre stars*). **b** Building fraction at 30-degree arcs extending 0.5 km from each station. **c** Mean UHA for the wind sector $\theta$ and station i ($\overline{{T}^{({\theta })}_{UHA(i)}})$, defined in detail below) in three wind-speed groups (WG1, *solid blue line*: ${<}2\,\hbox {m}\,\hbox {s}^{-1}$, WG2, *dashed green line*: 2–3$\,\hbox {m}\,\hbox {s}^{-1}$, WG3, *dotted red line*: ${>}3\,\hbox {m}\,\hbox {s}^{-1})$

Categorized land-cover products (Stathopoulou and Cartalis 2006), vegetation indices (Chen et al. 2006) or local climate zones (Stewart and Oke 2012) are frequently used to link the UHI magnitude to local land use. However, the resolution of such products is generally too coarse to study small-scale UHA features. As such, building fractions calculated from Ordnance Survey 1:10000 VectorMap data are used (a dataset that contains outlines of all individual buildings in the UK: see Fig. 2a for examples of the data at selected Met Office stations). The Ordnance Survey data were aggregated to 30-degree sectors within a 0.5-km radius to create a “directional building fraction” for each station (Fig. 2b). Based on Oke (2006), a 0.5-km radius was initially selected to represent the circle of influence for screen-level temperatures (a distance that has also been adopted for classifying local climate zones: Stewart and Oke 2012). Whilst the building-fraction methodology presented could be used as a simple means to provide enhanced urban metadata for station networks, it should be noted that the calculated building fraction will underestimate the actual urban fraction because only building footprint areas are available in the dataset (and not all other paved surfaces). This limitation should not significantly affect the outcome of a correlation analysis in Sect. 3 since an approximate proportionality between building fraction and urban fraction was assumed. However, this may not be the case in all urban areas worldwide, e.g. extensive paved areas in U.S. cities.

### Selection of Stations

The UK Met Office station network consists of approximately 200 operational weather and climate stations (Met Office 2016), where the exposure of each station is chosen to represent the weather and climate of a wide area ($\approx$40-km spacing). Data from this network were accessed through the Met Office Integrated Data Archive System that provides land-surface observations from 1853 to present (Met Office 2012). Air temperature (accurate to 0.1$\,^{\circ }\hbox {C}$) was measured at 1.25 m above the ground using thermometers located inside Stevenson screens, while wind data were collected at 10 m above ground (speed accurate to $0.51\,\hbox {m}\,\hbox {s}^{-1}$ and direction within $5^{\circ }$). Only stations with 1-h temperature and wind records covering a year or more were selected for further analysis. Next, digital elevation data from the Shuttle Radar Topography Mission (Reuter et al. 2007) were used to determine the local topography at each station. As in Kalnay and Cai (2003), albeit more conservative, our study only used stations located at altitudes less than 300 m. Additionally, stations with a significant local topography change (${>}150\,\hbox {m}$ in a 1-km radius) and stations located near coasts (${<}5\,\hbox {km}$) were excluded from the analysis. Although, sea breezes can extend beyond this distance, the analysis was limited to nighttime (see Sect. 2.4), therefore limiting any coastal effects. Overall, these measures were taken as an attempt to exclude unwanted advective warming or cooling.

**Fig. 3:**
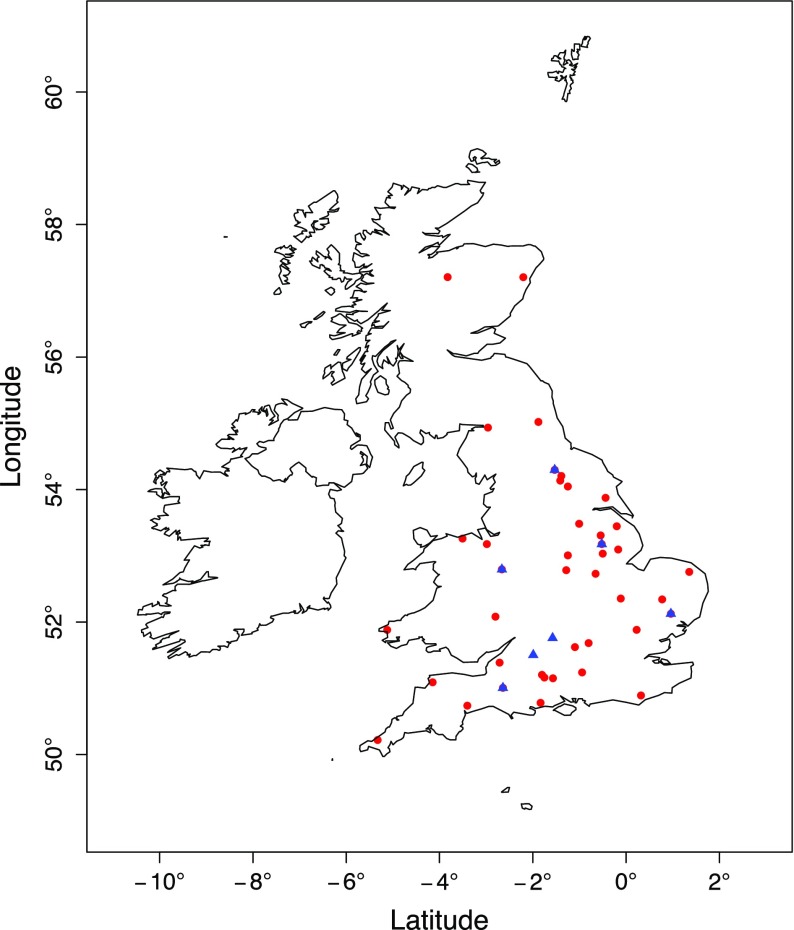
Stations used for the baseline temperature series (*blue triangles*) and advection analysis (*red dots*)

For the remaining stations that satisfied these conditions, there was a need to identify specific sites prone to urban heat advection for detailed analysis. Building on Lowry (1977), it was assumed that only stations within the urban environ will be influenced by urban heat advection under a given wind sector. To identify these stations a two-stage approach was taken. Firstly, the building fraction data were used to automatically exclude from the analysis: (i) rural stations with no local urbanization in the surrounding area (0.5-km radius), and (ii) urban stations (i.e. the land-use pattern would be too complex to separate local effects). Secondly, a visual check using satellite imagery was used to confirm the automatic station classification was correct (satellite imagery and urban fraction is presented in the Online Resource 1). This visual check highlighted further stations that needed to be excluded from the analysis that were either surrounded by larger scale urban features or were next to other external heat sources or sinks, e.g. lakes or coal power stations. Overall, 42 stations were identified with available 1-h temperature data at locations that could be considered at a high risk of urban heat advection (Fig. 3). Typically, sites were located adjacent to a village or small town with an area of approximately $1\,\hbox {km}^{2}$. Many sites in the analysis were weather stations located near small airfields, a consequence of the historical link between aviation and meteorology.

### UK Baseline Temperature

**Fig. 4:**
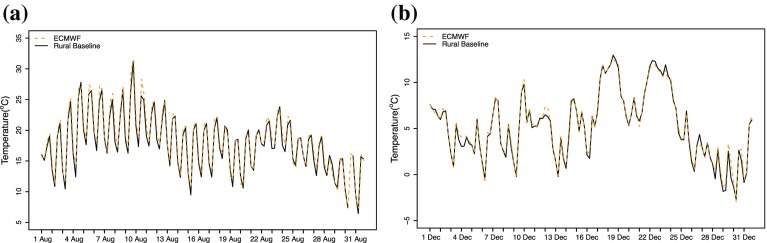
ECMWF ERA-Interim comparison with the baseline temperature series: **a** August 2003, and **b** December 2014

The temperature data across all available stations were not temporally homogeneous (stations may be re-sited or closed over time). To ensure reliable comparisons across stations when urban heat advection was calculated, a consistent baseline temperature series was required. The baseline covered a 30-year analysis period from 1985 to 2015 at a 1-h resolution. In the spatial domain, ideally, one ‘pure rural’ station (i.e. no surrounding urban heat sources) close enough to each of these 42 stations would be sufficient, but close scrutiny of the stations demonstrated that this was rarely available. In total, just seven suitable rural stations were found that had continuous temperature data (greater than 99% data capture) over the 30-year analysis period (Fig. 3). By adjusting for altitude (range of 20–145 m) using the environmental lapse rate (6.0$\,^{\circ }\hbox {C}\;\hbox {km}^{-1})$, the dominant control for mean temperature across the 30-year period was latitude ($R^{2}=0.93$). Based upon this, two different baseline temperature series were constructed from the data of the seven stations: (i) the mean temperature of these seven stations for each hour over the 30-year time period, denoted by $T_B(t)$, and (ii) latitude-dependent 1-h data over the 30-year time period, denoted by $T_B ({t, y})$. It will be demonstrated later in Sect. 2.4 that for the UHA analysis, use of the two types of baseline temperature series is identical under a reasonable assumption for the functional form of $T_B({t, y})$. Thus, $T_B(t)$ was adopted, which is a function of time (*t*) and independent of location. It was also acknowledged that whilst stations used in the baseline were considered rural, they could have contained unavoidable UHA influences. However, any impacts on results were limited by taking the mean of several stations to create the baseline. In order to check the representivity of the baseline, a comparison was made with ECMWF ERA-Interim (Balsamo et al. 2015) re-analysis temperature data. A grid point (52.125, −1.625) in the middle of the seven stations was chosen for the comparison, with data taken at 6-h intervals. At midnight (0000 UTC) the ECMWF reanalysis was found to strongly correlate with the baseline temperature series over the 30-year period ($R=0.99$, mean squared error = 0.4$\,^{\circ }\hbox {C}$). Example data from individual months are presented in Fig. 4 where it was evident that the baseline temperature series and ECMWF reanalysis follow the same trend. This provided evidence of the suitability for interpreting this baseline as a background climate for later use in the UHA calculation.

### Urban Heat Advection

This section demonstrates that the use of a location-independent 1-h rural baseline, $T_B({t_n})$, in the UHA analysis is justifiable, where $t_n$ denotes the *n*-th hour, and subscript *B* denotes ‘baseline’. As indicated in Sect. 2.3, analysis of the data from the seven rural stations’ showed that the dominant control in the spatial domain (longitude, latitude, altitude, or *x*, *y*, *z*) for temperature was latitude ($R^{2} = 0.93$). It is thus evident to assume a latitude-dependent baseline temperature time series, or even in a much more general sense, a location-dependent baseline temperature time series, $T_{B,i}({t_n , x_i ,y_i })$, which can be decomposed into two terms,

1 $$\begin{aligned} T_{B,i} \left( {t_n, x_i, y_i } \right) =T_B \left( {t_n )+T_B^{\prime } (x_i ,y_i } \right) , \end{aligned}$$

where subscript *i* denotes the *i*-th station, $x_i$ and $y_i$ are the longitudinal and latitudinal coordinates of the *i*-th station. $T_B^{\prime }({x_i ,y_i})$ represents regional-scale variability of $T_{B,i}$ (by definition it is an offset of $T_{B,i}$ from $T_B$), and was a function of location only. Analysis of the temperature data at the seven stations showed that the offset of $T_{B,i}({t_n, x_i, y_i })$ from $T_B ({t_n })$ were strongly dependent on latitude, $y_i$ (Sect. 2.3), and this provides evidence to support the assumption (1). However, this assumption does not include other effects that could become apparent with larger latitude changes, i.e. sunrise/sunset times.

The following demonstrates that use of a hypothetical location-independent $T_B ({t_n })$ and location-dependent baseline temperature time series $T_{B,i}({t_n, x_i, y_i})$ satisfying (1), yielded identical results for the directional UHA analysis described in Bassett et al. (2016). By denoting the observed temperature data at the *i*-th station as $T_i ({t_n })$, the increases in temperature at this site relative to the two baseline temperatures, $T_{B,i} ({t_n, x_i, y_i})$ and $T_B ({t_n})$, are,

2 $$\begin{aligned} \Delta _{B,i} T_i =T_i ({t_n })-T_{B,i} \left( {t_n, x_i, y_i } \right) , \end{aligned}$$

and

3 $$\begin{aligned} \Delta _B T_i =T_i ({t_n })-T_B ({t_n }), \end{aligned}$$

respectively. Equations 1–3 can be combined to obtain,

4 $$\begin{aligned} \Delta _{B,i} T_i =\Delta _B T_i -T_B^{\prime } \left( {x_i ,y_i } \right) . \end{aligned}$$

Applying the directional UHA analysis (Bassett et al. 2016), firstly the all-hour-mean (i.e. all wind directions included) warming increment, $\overline{\Delta _{B,i} T_i }$, from (4) was obtained,

5 $$\begin{aligned} \overline{\Delta _{B,i} T_i } =\overline{\Delta _B T_i } -T_B^{\prime } \left( {x_i ,y_i } \right) . \end{aligned}$$

Then the mean warming increment was calculated for one direction sector, $\theta$ (a 30-degree interval), from (4),

6 $$\begin{aligned} \overline{\Delta _{B,i} T_i^\theta } =\overline{\Delta _B T_i^\theta } -T_B^{\prime } \left( {x_i ,y_i } \right) . \end{aligned}$$

Finally, the all-hour-mean warming increment, $\overline{\Delta _{B,i} T_i }$, was subtracted from $\overline{\Delta _{B,i} T_i^\theta }$ to give the mean urban heat advection for the wind sector,

7 $$\begin{aligned} \overline{T_{UHA \left( i \right) }^{\left( \theta \right) } } =\overline{\Delta _{B,i} T_i^\theta } -\overline{\Delta _{B,i} T_i } . \end{aligned}$$

Following the substitutions of (5) and (6) into (7), this provides,

8 $$\begin{aligned} \overline{T_{UHA \left( i \right) }^{\left( \theta \right) } } =\overline{\Delta _B T_i^\theta } -\overline{\Delta _B T_i } . \end{aligned}$$

The interpretation of the notations in (7) and (8) was that (7) calculates the mean urban heat advection for the wind sector $\theta$ using location-dependent baseline temperature time series, $T_{B,i} \left( {t_n, x_i ,y_i } \right)$, whereas (8) calculates the same quantity using location-independent baseline temperature time series, $T_B ({t_n })$. Thus, this demonstrates that the use of $T_B ({t_n })$ and use of $T_{B,i} \left( {t_n, x_i, y_i } \right)$ yield identical results for the directional UHA analysis; the condition is that assumption (1) holds.

Through prior UHA studies (Heaviside et al. 2015; Bassett et al. 2016), a positive mean UHA value ($\overline{T_{UHA \left( i \right) }^{\left( \theta \right) }})$ is expected if a given station is located downwind of an urban area. Due to the way $\overline{T_{UHA \left( i \right) }^{\left( \theta \right) }}$ is defined, negative results indicate that the temperature in a given wind sector is less than the all-hour mean. In reality, a range of air-mass types have different stability conditions, which would affect the results. To account for directional stability inhomogeneity, data corresponding to nighttime (from sunset to sunrise) and low cloud cover (<5 oktas) conditions were selected. Additionally the data were classified into three wind-speed groups (WG1: ${<}2\,\hbox {m}\,\hbox {s}^{-1}$, WG2: $2 - 3\,\hbox {m}\,\hbox {s}^{-1}$, WG3 $>\,\,3\,\hbox {m}\,\hbox {s}^{-1})$. These categories, applied to all results, represent the Pasquill-Gifford stability (Pasquill and Smith 1983) conditions from neutral to stable, where conditions have been shown to favour large UHI magnitudes (Tomlinson et al. 2012; Azevedo et al. 2016).

## Results and Discussion

### Case Studies

**Fig. 5:**
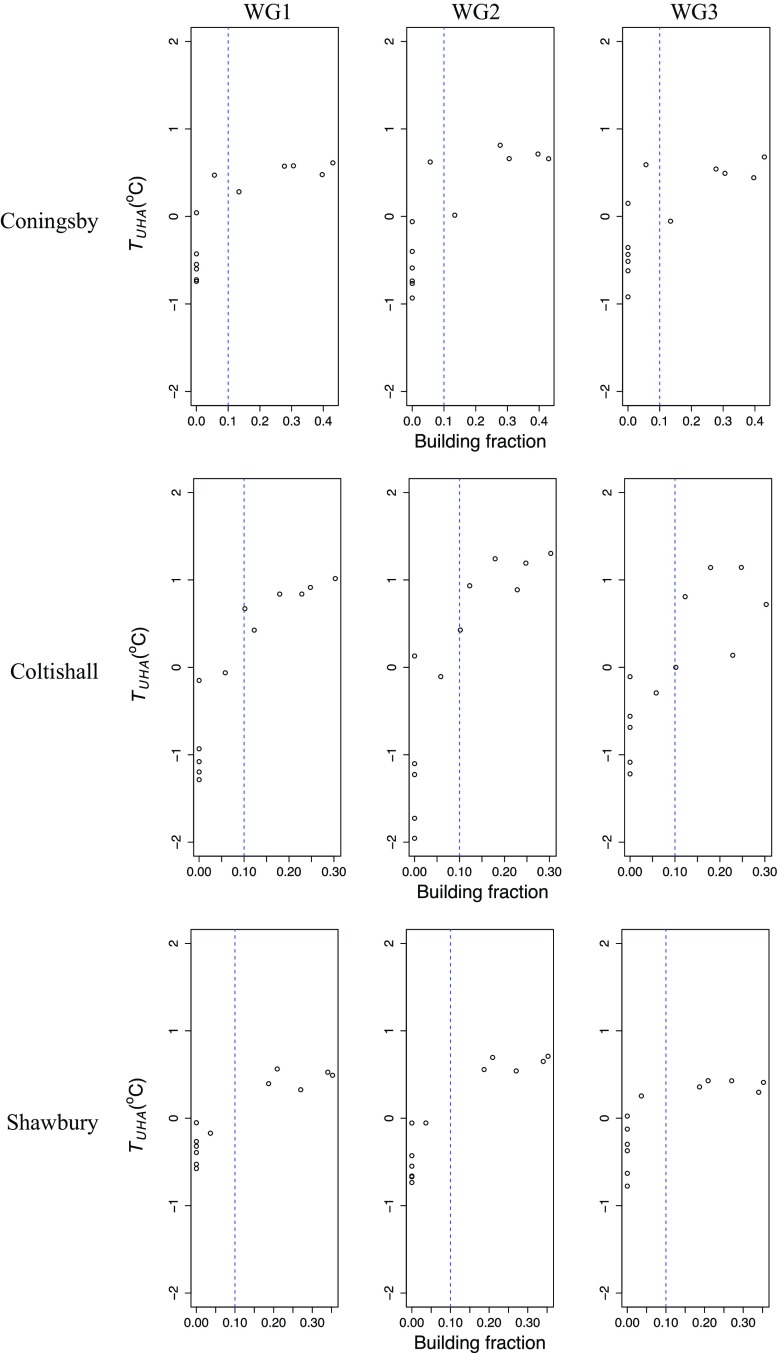
The relationship between 0.5-km upwind building fraction and mean UHA ($\overline{T_{UHA (i)}^{(\theta )} } )$ in three wind-speed groups at Coningsby, Coltishall and Shawbury weather stations (WG1: $<\,2\,\hbox {m}\,\hbox {s}^{-1}$, WG2: $2 - 3\,\hbox {m}\,\hbox {s}^{-1}$, WG3: $>\,3\,\hbox {m}\,\hbox {s}^{-1})$

In order to demonstrate the methodology, three case studies are presented for the Met Office stations at Coningsby, Coltishall and Shawbury (Fig. 2). Each station is consistent with the criteria for identifying stations suitable for analysis: flat terrain, inland from the coastline and a simple urban pattern in a single direction (Fig. 2a). The calculated upwind building fraction at 30-degree intervals extending to 0.5 km from the station for each station is presented in Fig. 2b. The mean directional temperature anomaly or urban heat advection ($\overline{T_{UHA (i)}^{(\theta )}})$ for each case study (Fig. 2c) indicates a bias in the same direction as the urban area next to the station. At each station a clear relationship is found in all wind-speed groups whereby $\overline{T_{UHA (i)}^{(\theta )}}$ is linked to the upwind urban fraction. At Shawbury, the difference between the mean $\overline{T_{UHA (i)}^{(\theta )}}$ for all urban sectors (i.e. for $\theta$ with building fraction >0.1) and for all rural sectors ($\theta$ with building fraction <0.1) is 1.1$\,^{\circ }\hbox {C}$. For Coningsby and Coltishall, the mean $\overline{T_{UHA (i)}^{(\theta )}}$ for all urban sectors is up to 1$\,^{\circ }\hbox {C}$ and 2$\,^{\circ }\hbox {C}$ greater than rural sectors respectively. A scatterplot showing the relationship between the upwind building fraction and temperature anomaly at these stations is presented in Fig. 5. Whilst these UHA values seem large, mesoscale modelling, albeit on a different scale, has shown similar values of urban heat advection to be plausible (Heaviside et al. 2015).

### Urban Heat Advection

**Fig. 6:**
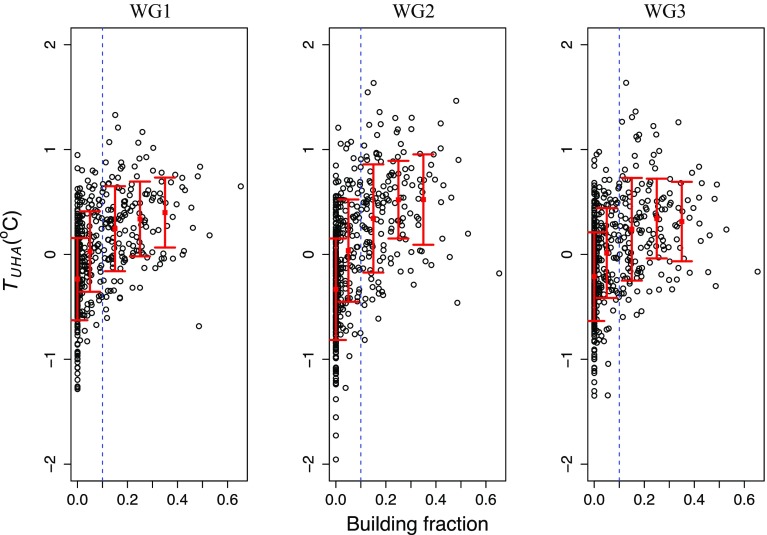
The relationship between upwind building fraction and the mean UHA signal ($\overline{T_{UHA (i)}^{(\theta )}})$ across all stations in three wind-speed groups (WG1: $<2\,\hbox {m}\,\hbox {s}^{-1}$, WG2: 2–3$\,\hbox {m}\,\hbox {s}^{-1}$, WG3: $>3\,\hbox {m}\,\hbox {s}^{-1})$. The *red square* indicates the mean urban heat advection at urban fractions: 0, 0–0.1, 0.1–0.2, 0.2–0.3, 0.3–0.4. The *vertical lines* either side of the mean represent ± one standard deviation. The *dashed blue line* at the 0.1 building fractions indicates the urban, rural separation used for statistical analysis in Table 1

The analysis was then re-scaled to include each station (42 total). Figure 6 shows the relationship between upwind building fraction and the urban heat advection ${\overline{(T_{UHA (i)}^{(\theta )})}}$ across all stations, including the mean urban heat advection and standard deviations over 0.1 building fraction intervals. The mean urban heat advection incrementally increases with each increase in building fraction, from zero to 0.3, before generally reaching a plateau, likely caused by fewer data points available at dense urban areas (for this reason the mean and standard deviation are not shown at building fraction intervals >0.4).

To test the significance of UHA ($\overline{T_{UHA (i)}^{(\theta )}})$ differences between wind sectors, the data are split into two groups by their upwind building fractions (for $\theta$ in the urban sectors, building fraction >0.1 and $\theta$ in the rural sectors, building fraction <0.1). These groups contain 154 and 350 values of $\overline{T_{UHA (i)}^{(\theta )}}$ respectively. A null hypothesis is created stating that: “there is no significant difference between the temperature anomaly caused by urban and non-urban wind sectors”. The data are tested for consistency with a normal distribution (skewness between −1 and 1) and Welch’s two-sample *t*-test is conducted to determine whether there is a significant difference between means. The results of the *t*-test are summarized in Table 1. For each wind-speed group, a significance *p* value < 0.001 is calculated, thus the null hypothesis can be rejected. It can be concluded that increased temperatures for the airflow from urban sectors, relative to the flow from rural sectors, are due to the change in upwind characteristics from rural to urban. The difference between the mean of all urban sectors ($\overline{T_{UHA}^{({urban})} } )$ and the mean of all rural sectors ($\overline{T_{UHA}^{({rural})}})$ shows that the effect of upwind urban sectors contributes to a mean air-temperature increase of 0.6$\,^{\circ }\hbox {C}$ under low cloud cover at night (WG2). Whilst this warming may not be considered large, it is important to note these are averaged effects and therefore may be larger on individual nights at given stations. Indeed, already the case studies presented in Sect. 3.1 have demonstrated that a significantly larger UHA signal is possible. A distance analysis is conducted in the next section to confirm whether this 0.5-km radius used is appropriate. Of 42 stations analyzed, 32% of the wind sectors are considered urban (>0.1 building fraction), and assuming equal distribution of airflow in all wind directions, the overall mean nocturnal warming under low cloud cover is therefore 0.1$\,^{\circ }\hbox {C}, 0.2\,^{\circ }\hbox {C}$ and 0.2$\,^{\circ }\hbox {C}$, in WG1, WG2 and WG3 respectively.

**Table 1:** Welch’s two-sample *t*-test between $\overline{T_{UHA (i)}^{(\theta )} }$ for urban and rural wind sectors where the urban sectors correspond to $\theta$ with building fraction >0.1 and the rural sectors to $\theta$ with building fraction <0.1. The mean of all urban sectors is shown as $\overline{T_{UHA}^{({urban})}}$ and for rural sectors as $\overline{T_{UHA}^{\left( {rural} \right) } }$

	*t*-value	Degrees of freedom	*p* value	$\overline{T_{UHA}^{({rural})}} (^{\circ }\hbox {C}$)	$\overline{T_{UHA}^{({urban})}}(^{\circ }\hbox {C}$)	$\overline{T_{UHA}^{({urban})} } -\overline{T_{UHA}^{({rural})}}(^{\circ }\hbox {C}$)
WG1	11.5	306.2	<0.001	$-$0.13	0.30	0.43
WG2	13.51	312.3	<0.001	$-$0.19	0.43	0.62
WG3	9.7	291.8	<0.001	$-$0.13	0.28	0.41

A large degree of scatter is found within the data (Fig. 6). Indeed, relating an upwind land-use parameter to a meteorological observation is not straightforward. The scatter in the UHA signals may be attributed to the building-fraction methodology: (i) building fraction represents building coverage, or roof area and not the total urban fraction, (ii) the size and complexity of each adjacent urban sector differs, and (iii) the form and function of each building is not considered. This makes analysis challenging because for a given wind category, no two stations necessarily have the same upwind urban land use. In addition, if there are two stations with the same building fraction profiles, flow and turbulent characteristics within allocated groups may differ due to other unaccounted local or regional factors. The location of the buildings within the 0.5-km wind sector may also differ between two stations that are classified with identical 30-degree upwind building fractions. Whilst the use of Ordnance Survey building data has its limitations, this sub-km analysis is not possible with coarser resolution products typically used to classify the UHI (e.g. satellite data).

Whilst the overall pattern shows a significant UHA effect, and most stations exhibit a pattern similar to that presented in the case studies (Fig. 2), a handful of stations within the dataset do not follow this trend. These discrepancies can be readily explained (Table 2) when interpreting satellite imagery for the given station (Online Resource 1). In addition, the mean UHA signal at several stations only contains a relationship with building fraction in one or two of the wind-speed groups. This may be explained by station specific characteristics that are not accounted for, through simply classification of the data using urban fraction and stability conditions. The explanations provided highlight the previously discussed limitations in the building-fraction methodology, as opposed to a lack of a UHA signal. The methodology also does not account for changes in urbanization over the 30-year analysis period since building fraction was calculated at a fixed point in time. Additionally, all paved surfaces that could generate urban heat advection are not presently accounted for and the distance at which each station is located from the urban areas varies. There is also a large range of (negative) UHA values associated with rural (zero building fraction) directions. The methodology is presently unable to determine vegetation type (e.g. open fields or forests) or other external heat sources that could account for these differences. Vegetation could also help explain UHA scatter for upwind urban directions.

**Table 2:** Stations where the UHA signal does not exhibit a relationship with upwind building fraction with explanations provided through a visual station analysis

Station (ID)	Explanation
Binkbrook (394)	The station closed in 1992. The adjoining land use has since changed from an airfield to industrial estate. Therefore the calculated building fraction is different during the observation period
Herstmonceux (811)	Tall hedges surround the station on two sides, and slight elevation changes present nearby
Larkhill (888)	There is a visual link between the land use and UHA pattern, however this is not reflected in the building fraction data. For example south-west of the station there is a large area of paved surfaces. Additionally this station could have two UHA sources, therefore the pattern is less clear
Bristol/Lulsgate (18912)	The UHA pattern relates to satellite imagery, however similarly to Larkhill the building fraction does not capture a large portion of paved surfaces, in particular the airport car park

Under the same conditions (WG2, nighttime and cloud cover <5 oktas) that a mean difference between urban and rural wind sectors ($\overline{T_{UHA}^{({urban})} } -\overline{T_{UHA}^{({rural})} } )$ of 0.6$\,^{\circ }\hbox {C}$ is found, Bassett et al. (2016) observed a mean UHA signal of 0.4$\,^{\circ }\hbox {C}$ for Birmingham, UK. This was calculated by taking the mean UHA signal from a network of urban canopy stations downwind of the city centre. Whilst the mean urban heat advection found is over 50% greater than that found in Birmingham (a city several orders of magnitude larger in size than the urbanized areas in this study), conducting UHA analysis within a large city has several limitations: (i) the mean observed signal will be weakened by urban heat advection in opposing directions, (ii) a central urban reference station was used for the UHA calculation that could itself be influenced by local heat advection, and (iii) stations downwind of the central business district could be encircled by suburban land use (although analysis was still possible because certain upwind sectors, i.e. towards the city centre, contained higher urban fractions). By only analyzing stations with a clear distinction between urban and rural wind sectors, this could explain the higher observed UHA effect from small urbanized areas than that found in Bassett et al. (2016). The results also indicate the highest urban heat advection to be present under medium wind speeds ($\hbox {WG2:}$ 2–3 $\hbox {m}\,\hbox {s}^{-1})$, which is consistent with other UHA studies ( Brandsma et al. 2003; Bassett et al. 2016). At low wind speeds (WG1), whilst the UHI is most pronounced, there is little potential to transport heat. At high wind speeds (WG3) heat transport is increased, however the UHI is least pronounced. Therefore a balance between these factors occurs under medium wind speeds (WG2) leading to the highest UHA values. However even under WG1, air travels further than the distance between the station and urban boundary at the 1-h time scale.

**Table 3:** Pearson’s correlation coefficient between the mean UHA signal ($\overline{T_{UHA (i)}^{(\theta )} } )$ and upwind building fraction arcs at increasing distances from stations

	0.5 km	0.5–1 km	1–2 km	2–3 km
WG1	0.46	0.30	0.15	0.19
WG2	0.49	0.32	0.17	0.17
WG3	0.38	0.23	0.17	0.14

### Urban Heat-Advection Footprint

The results presented in Sect. 3.2 use a fixed building fraction distance of 0.5 km, as the suggested circle of influence on screen-level temperatures (Oke 2006). However UHA signals have previously been found at greater distances downwind (Brandsma et al. 2003; Bassett et al. 2016). To test the 0.5-km circle of influence, the methodology used to calculate building fraction is extended for 30-degree arcs extending: 0.5–1 km, 1–2 km and 2–3 km from each station. The building fraction calculated at these distances can be visualised as arcs, and do not contain the information from preceding (smaller) distances. Pearson’s correlation coefficient is calculated between the directional building fraction and the mean UHA signal ($\overline{T_{UHA (i)}^{(\theta )}})$ at these distances, with results presented in Table 3. The correlation coefficient is shown to reduce with increased distance from the stations. Whilst the results support Oke’s (2006) circle of influence with the largest correlation coefficients found at 0.5 km, the UHA footprint or distance is not directly comparable to other studies because different urban sizes are analyzed. Only the UHA effect from villages and small towns are considered, whereas Brandsma et al. (2003) and Bassett et al. (2016) analyzed temperature observations from large towns and cities. As such Brandsma et al. (2003) found distances in the order of several kilometres and Bassett et al. (2016) found evidence for urban heat advection at distances ${>}10\,\hbox {km}$. As observations from urban areas of different sizes are analyzed the processes and scale of UHA transport will differ considerably. Thus, the peak UHA correlation with upwind distance of 0.5 km may be an artefact of the length scale of buildings adjacent to the stations. Therefore, further work is needed to explore urban heat advection, perhaps in the form of downwind transects from urban areas of different sizes and complexities.

## Conclusions

Whilst the effects of large urbanization on station temperature data have previously been noted, urban heat advection, particularly from small urban areas, has rarely been considered. In total, 42 stations from the UK Met Office network were identified as having an adjacent urban area (approximately $1\,\hbox {km}^{2}$ size) in a single wind sector. The stations are typically located at airfields due to historical associations between aviation and meteorology, although these selected stations should not be considered an exclusive list of those likely to be influenced by urban heat advection. Station data with surrounding urban land use in all directions, near coasts or in areas of high terrain, were not analyzed. In these cases station data could also be affected by urban heat advection but the effect would be difficult to determine. In addition 1-h data were required, and with a large percentage of UK stations capturing only daily data, this limits the numbers of stations available for analysis.

Overall, the results demonstrate that even small urban areas ($\sim$ $1\,\hbox {km}^{2})$ exert a significant warming on their surroundings. An increase in air temperature between rural and urban upwind sectors of up to 0.6$\,^{\circ }\hbox {C}$ was found at night under low cloud cover and wind speeds of 2–3 $\hbox {m}\,\hbox {s}^{-1}$. This warming is larger than found in previous UHA observation studies. The warming presented due to urban heat advection is the mean pattern, and so may be larger on individual nights or at given stations (e.g. a 1.1$\,^{\circ }\hbox {C}$ difference at Shawbury). A degree of scatter was often found in the results and, indeed, several stations do not show any association between the UHA temperature anomaly and upstream building fraction. This may be associated with limitations in the building-fraction methodology, i.e. it does not represent the total urban fraction. However, for stations that do not follow the overall trend, a visual analysis using satellite imagery is often able to explain differences. The distance or circle of influence at which urban heat advection has the greatest effect was also tested finding building fraction within a 0.5-km distance to have the greatest influence on temperatures. This distance is in line with previous theory (Oke 2006) but does not match previous UHA studies that find greater distances (Brandsma et al. 2003; Bassett et al. 2016). However, these studies are not directly comparable because of scale and siting differences.

Station temperature data are crucial to comprehending trends in weather and climate. Although rigorous observational standards exist, our results question the representativeness of station siting, particularly for nighttime minimum temperatures taken near urban areas. Out of the 42 Met Office stations analyzed, 33 remain operational and therefore potentially contain a UHA bias. The traditional UHI methodology of calculating the temperature difference between an urban and rural station may also be scrutinized. The results demonstrate that a downwind rural reference station, even in an area outside of the main urban areas, may be influenced by local urban heat advection, and so underestimating the total UHI effect.

## Electronic supplementary material

Below is the link to the electronic supplementary material.

Supplementary material 1 (pdf 5037 KB)
