# Supplementary material for: The Effects of Heat Advection on UK Weather and Climate Observations in the Vicinity of Small Urbanized Areas
Source: Boundary Layer Meteorol. 2017 Jun 12;165(1):181–96. doi: 10.1007/s10546-017-0263-0 (PMC6961505; doi:10.1007/s10546-017-0263-0)
Supplement: Supplementary file 1 — Supplementary material 1 (pdf 5037 KB) [file 10546_2017_263_MOESM1_ESM.pdf]

Online Resource 1 Station metadata (accessed through the Met Office Integrated Data Archive System that provides UK land-surface observations from 1853 to present). In total, 42 stations from the UK Met Office network were identified as having an adjacent urban area (approximately 1 km^2 size) in a single wind sector. The stations are typically located at airfields due to historical associations between aviation and meteorology, although these selected stations should not be considered an exclusive list of those likely to be influenced by UHA. Station data with surrounding urban land use in all directions, near coasts or in areas of high terrain, were not analyzed. In these cases station data could also be affected by UHA but the effect would be difficult to determine. In addition 1-hr data were required, and with a large percentage of UK stations capturing only daily data, this limits the numbers available for analysis. The satellite imagery was used to visually check the automatic building fraction classification.

<table>
<tr><th>Station name (ID)</th><th>Latitude, Longitude</th><th>Building fraction at 30-degree arcs extending 0.5 km from each station</th><th>Satellite imagery of stations (Google Maps 2016)</th></tr>
<tr><td>Dyce (161)</td><td>57.2051, -2.2037</td><td>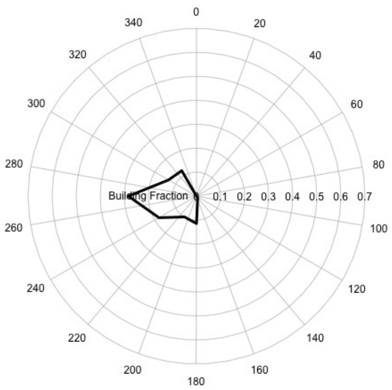
</td><td>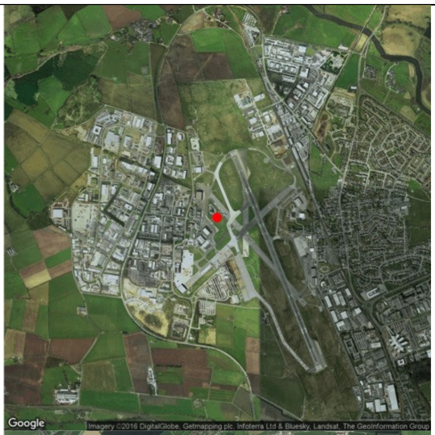
</td></tr>
<tr><td>Dishforth Airfield (342)</td><td>54.1346, -1.41293</td><td>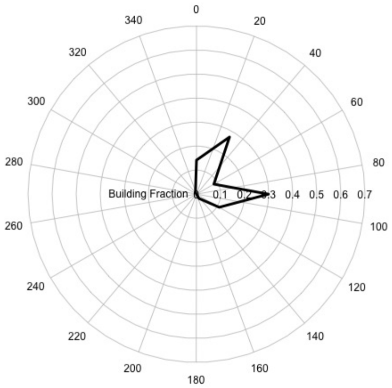
</td><td>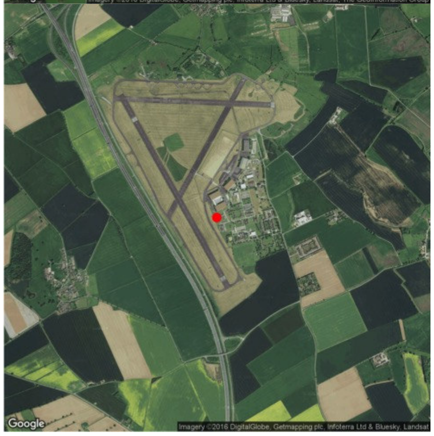
</td></tr>
</table>

Linton-on-Ouse (346)	54.045, -1.24956	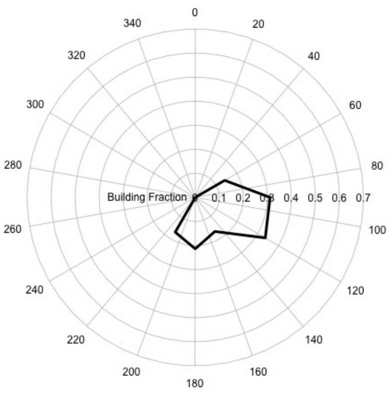	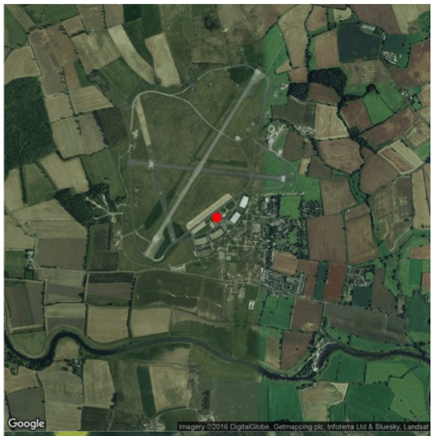
Leconfield (370)	53.8744, -0.44009	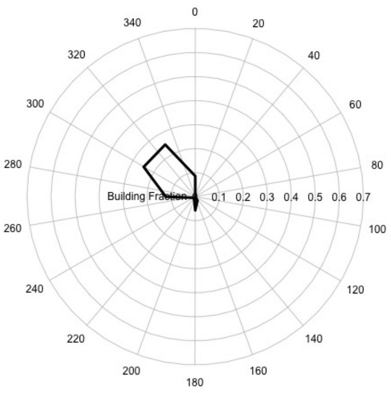	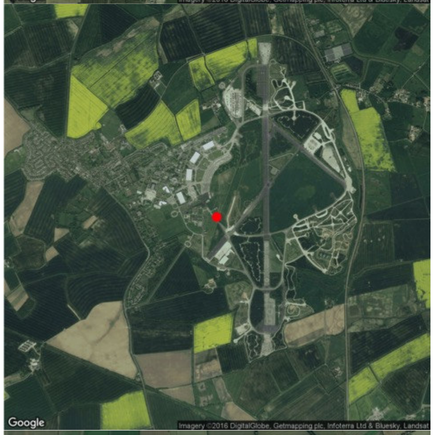
Scampton (381)	53.3066, -0.54649	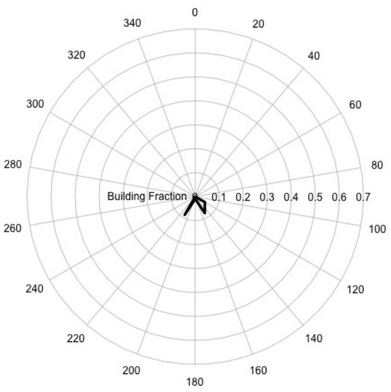	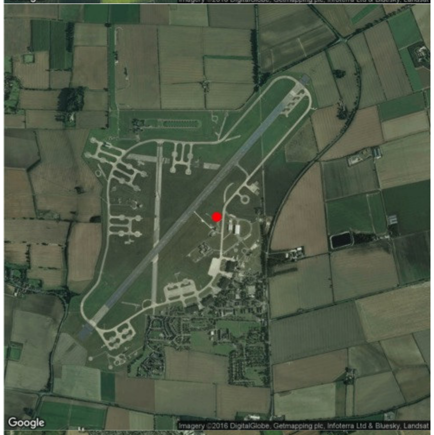
Waddington (384)	53.1751, -0.52173	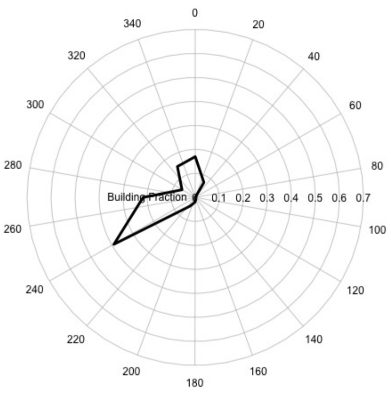	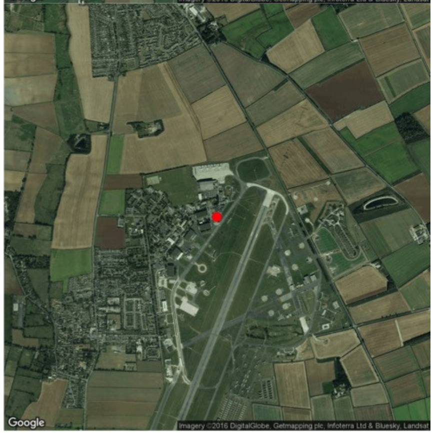

Cranwell (386) — 53.0309, -0.50194

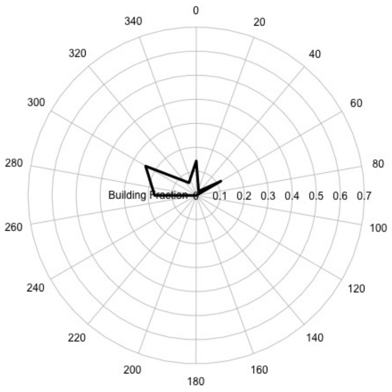

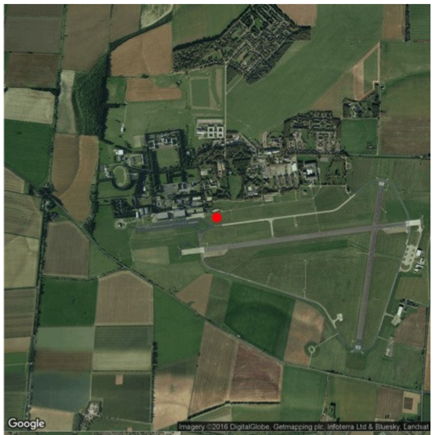

Coningsby (393) — 53.0935, -0.17119

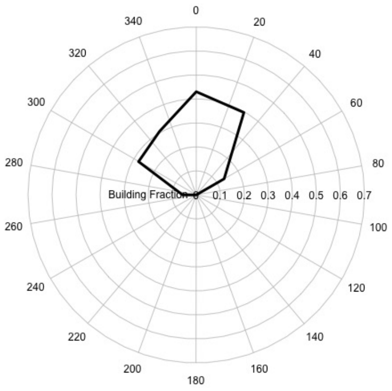

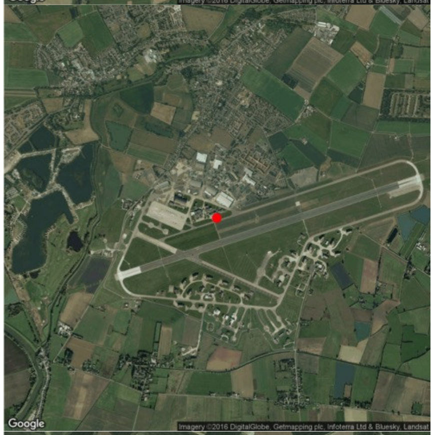

Binbrook (394) — 53.4451, -0.20053

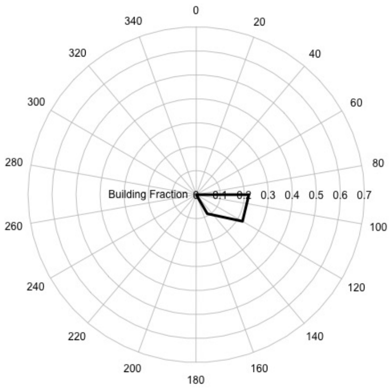

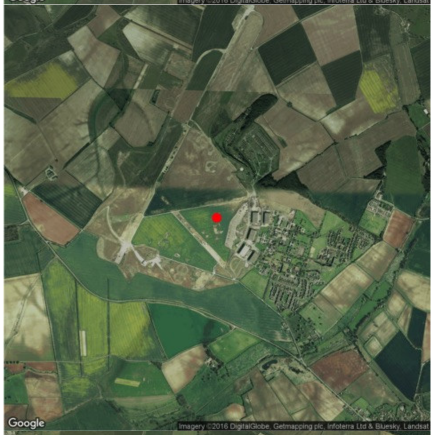

Marham (409) — 52.651, 0.56772

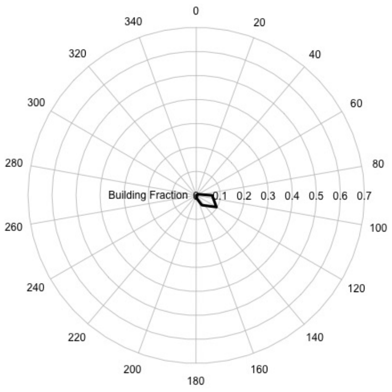

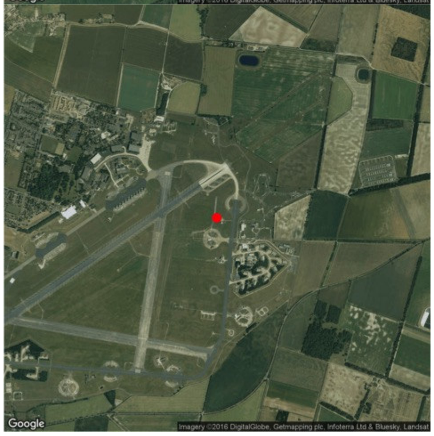

Coltishall (429)	52.7563, 1.35321	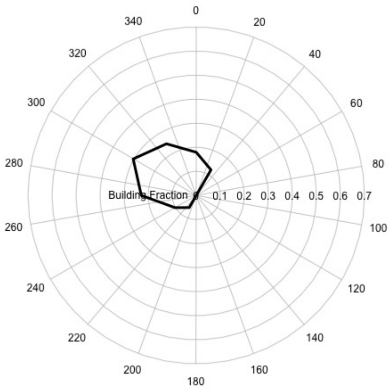	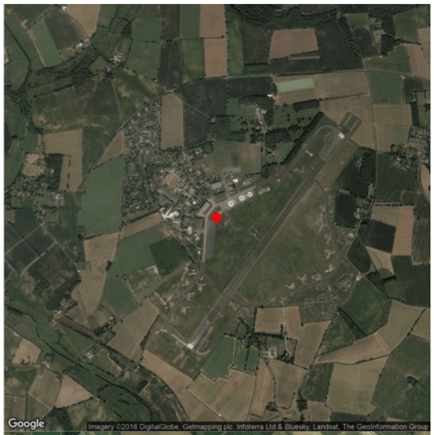
Honington (438)	52.3400, 0.77203	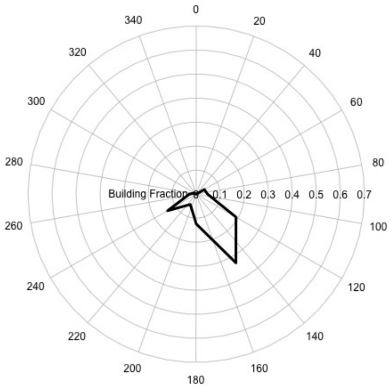	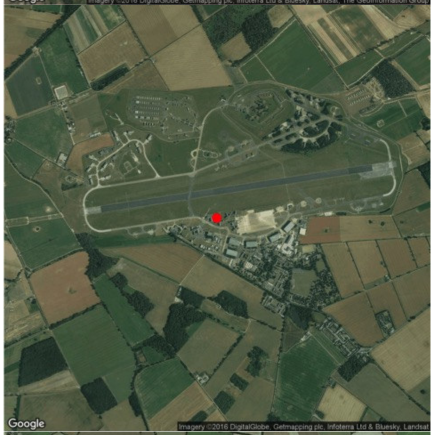
Wattisham (440)	52.1234, 0.9591	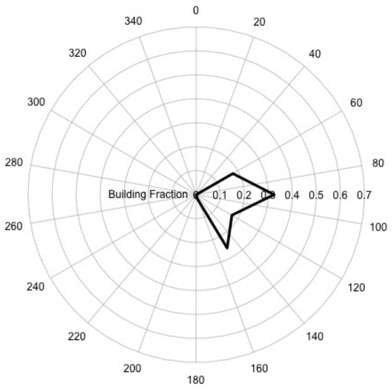	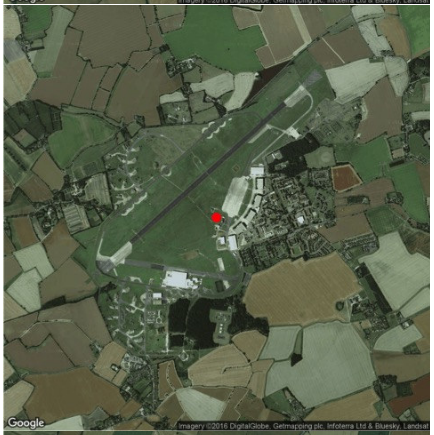
Wyton (457)	52.3531, -0.11452	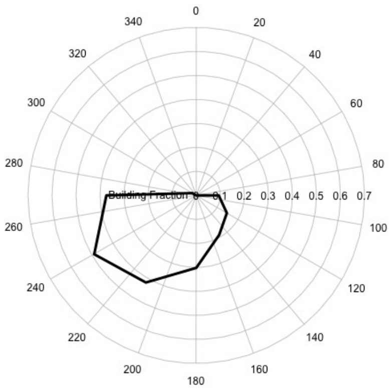	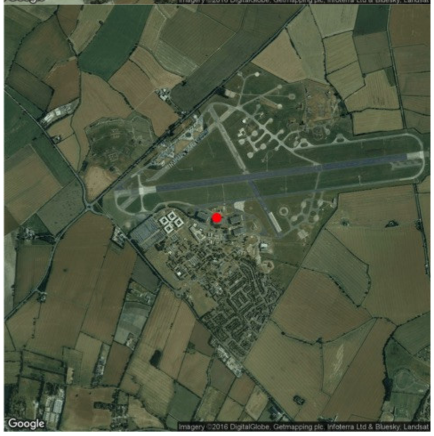

Stansted (484)

51.8805, 0.22456

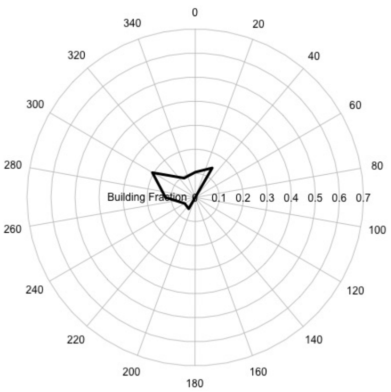

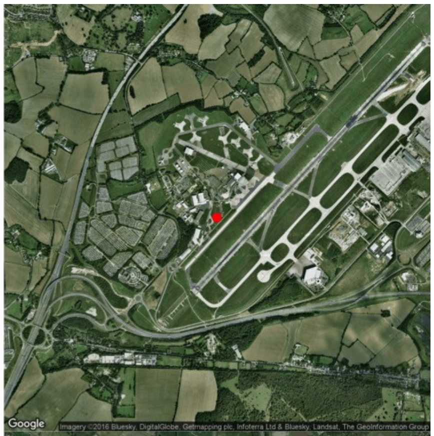

Nottingham Watnall (556)

53.0053, -1.24969

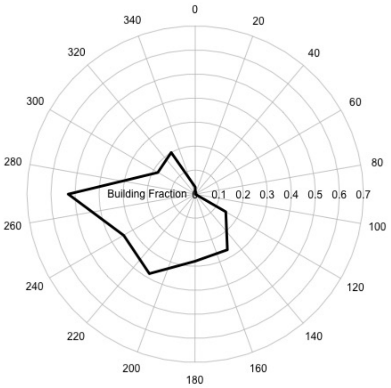

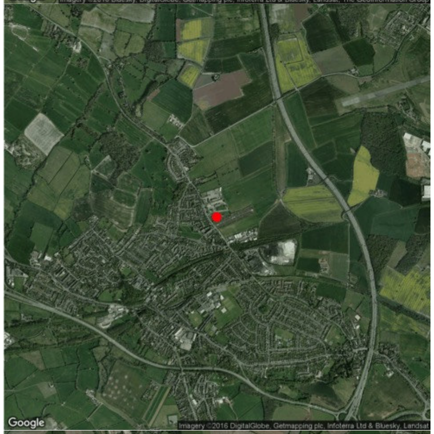

Finningley (562)

53.4824, -1.00682

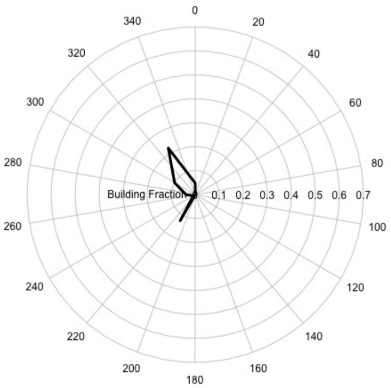

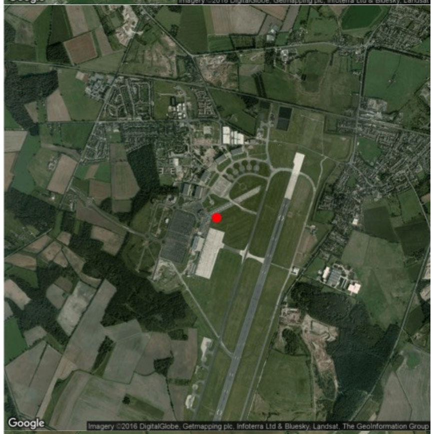

Cottesmore (576)

52.7270, -0.65439

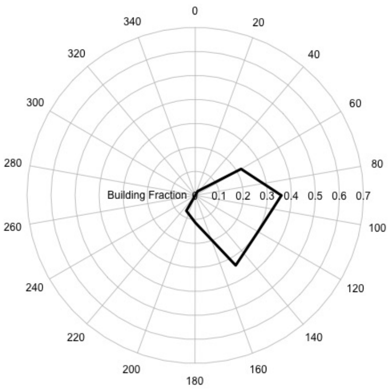

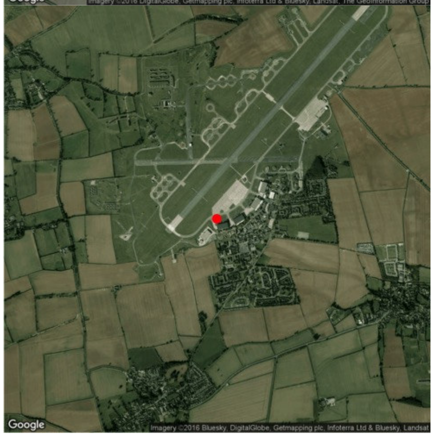

Coventry Airport (600)

52.3653, -1.48886

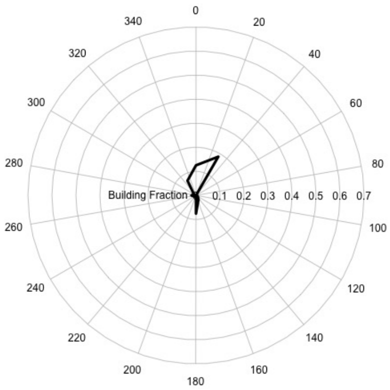

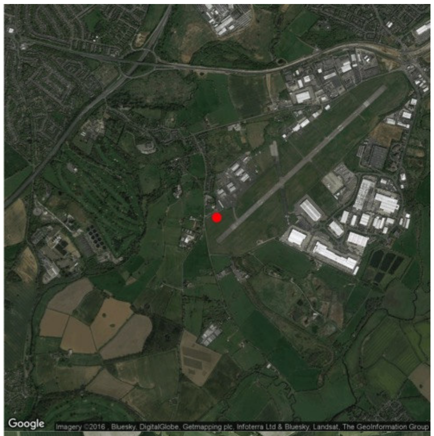

Benson (613)

51.6199, -1.09712

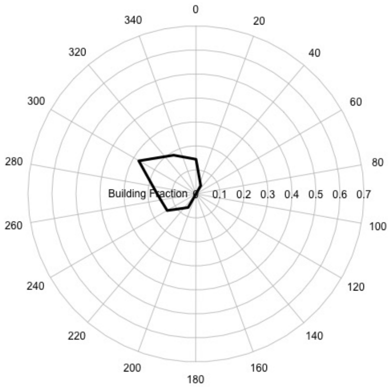

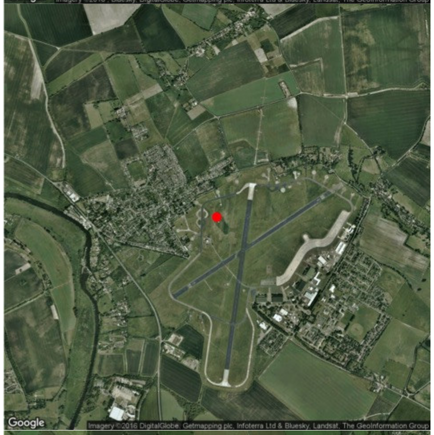

Shawbury (643)

52.7943, -2.66329

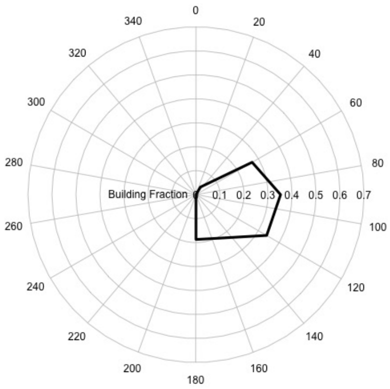

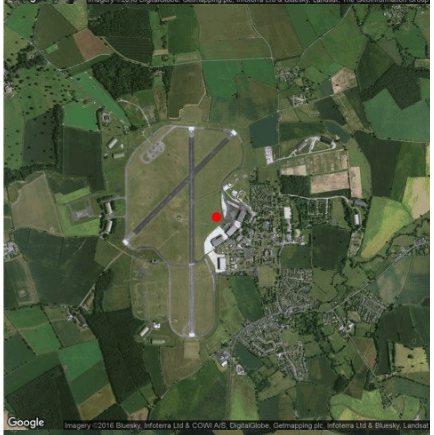

Pershore (657)

52.148, -2.03979

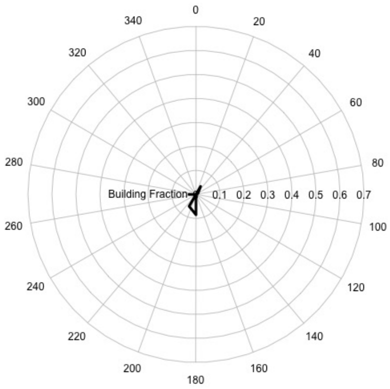

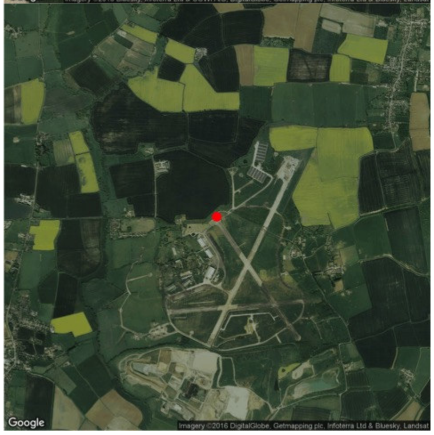

Herstmonceux West End (811)

50.8904, 0.31818

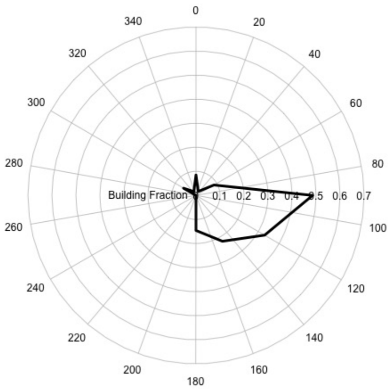

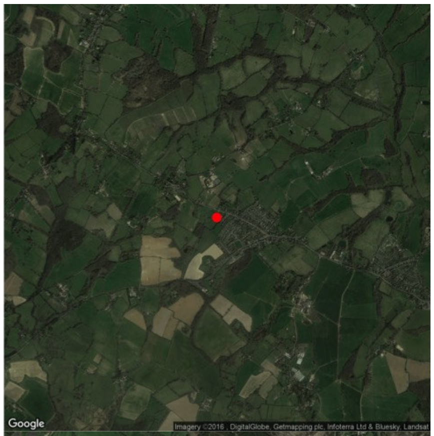

Hurn (842)

50.7789, -1.83483

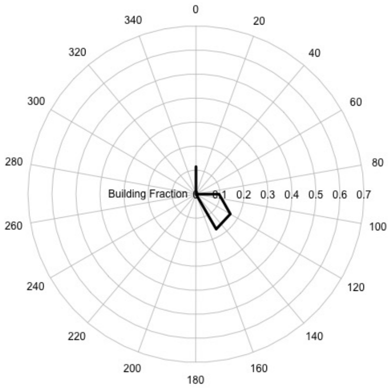

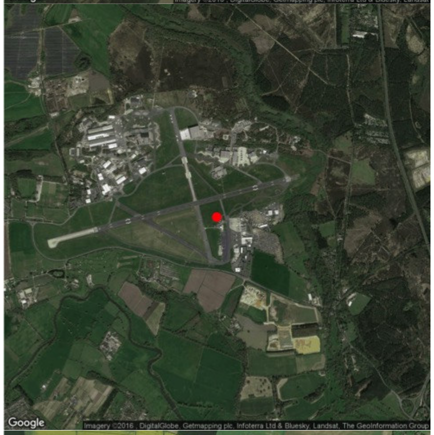

Middle Wallop (847)

51.1493, -1.56851

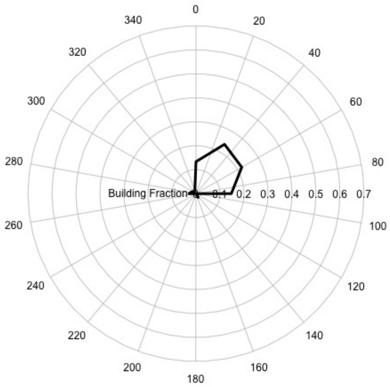

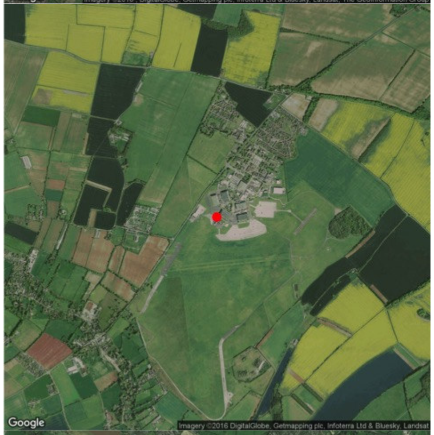

Odiham (862)

51.2385, -0.94346

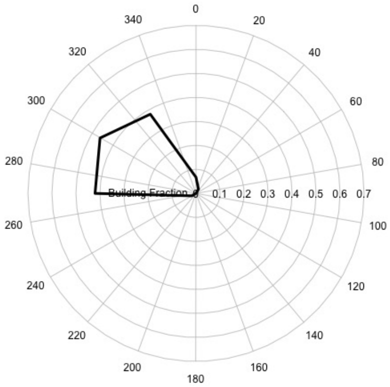

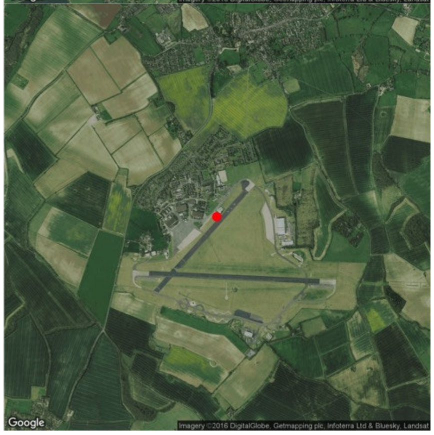

Larkhill (888)

51.2012, -1.80443

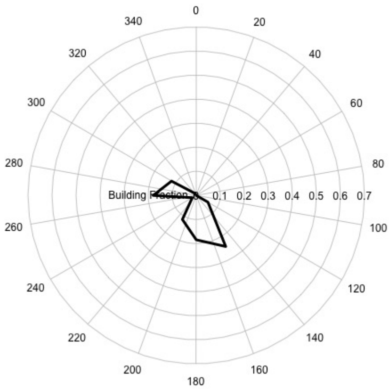

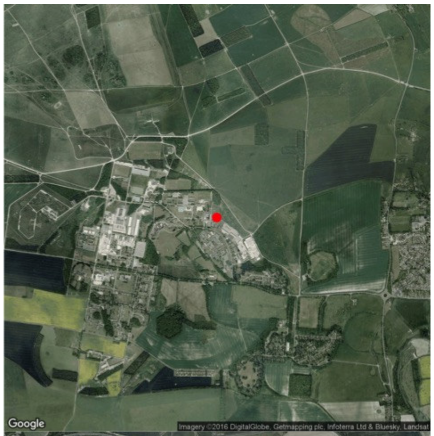

Boscombe Down (889)

51.1613, -1.75317

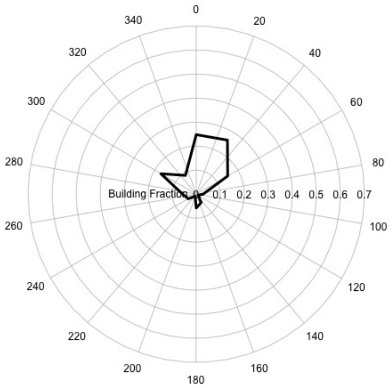

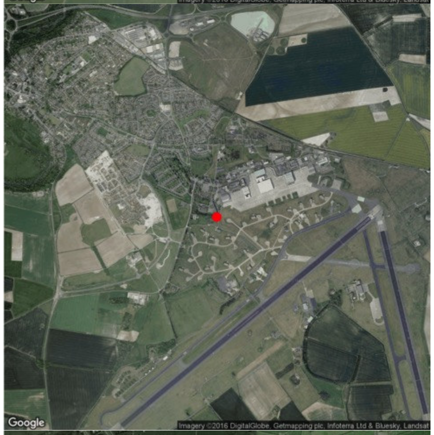

Carlisle (1070)

54.9342, -2.96223

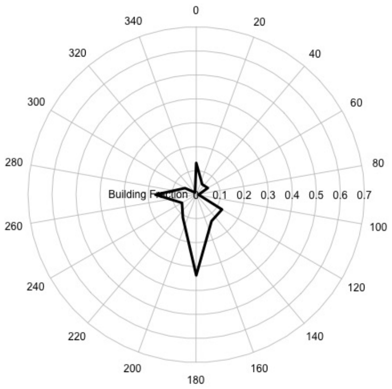

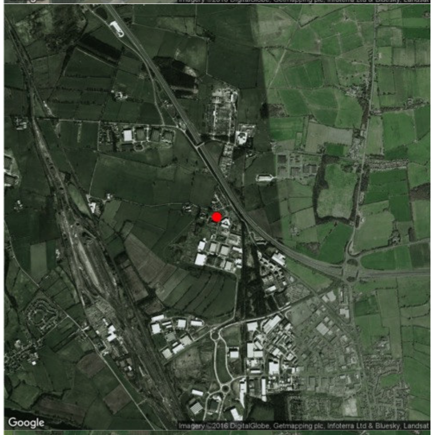

Rhyl No. 2 (1137)

53.259, -3.50754

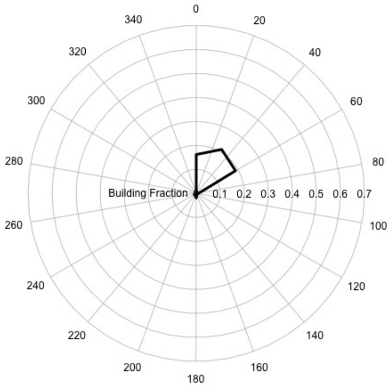

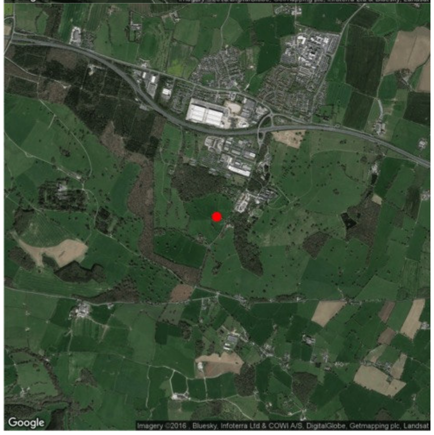

Hawarden Airport (1144)

53.1752, -2.98499

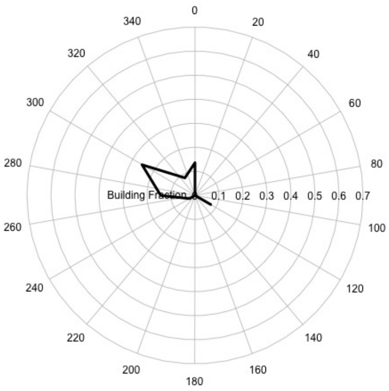

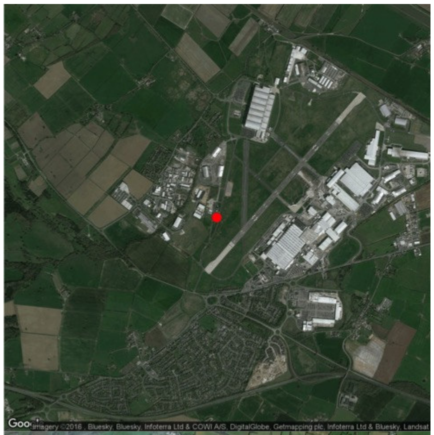

Yeovilton (1302)

51.0059, -2.64148

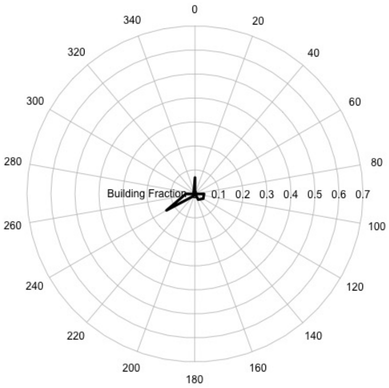

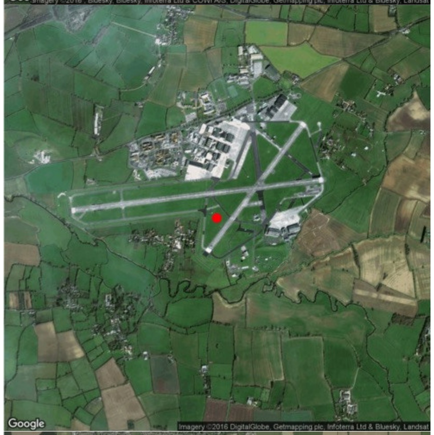

Chivenor (1346)

51.0886, -4.14743

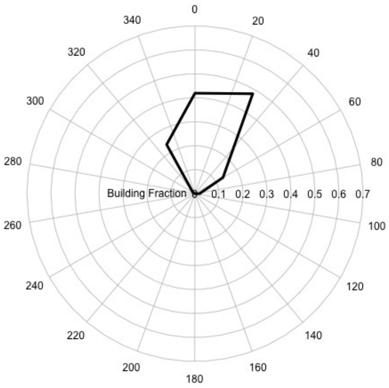

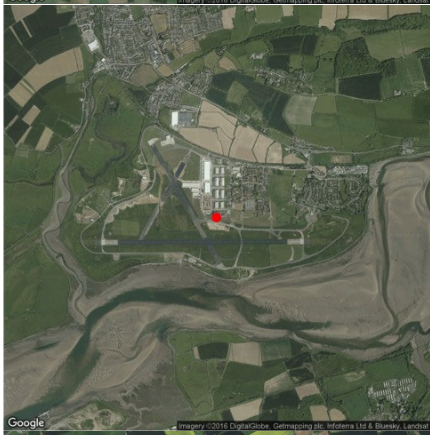

Topcliffe (16596)

54.2045, -1.38856

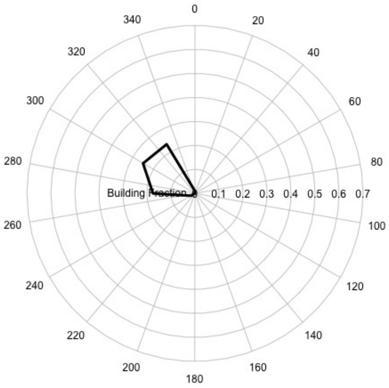

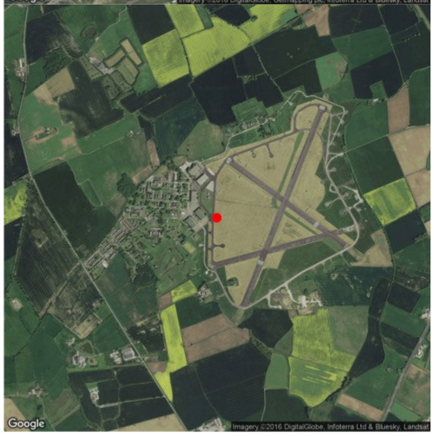

High Wycombe HQAIR (17176)	51.6813, -0.80528	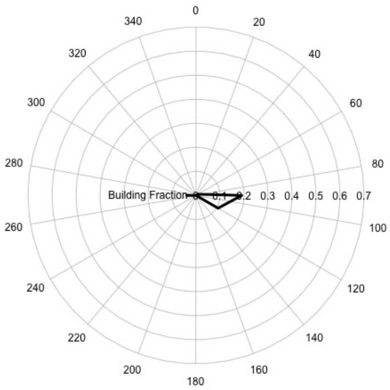	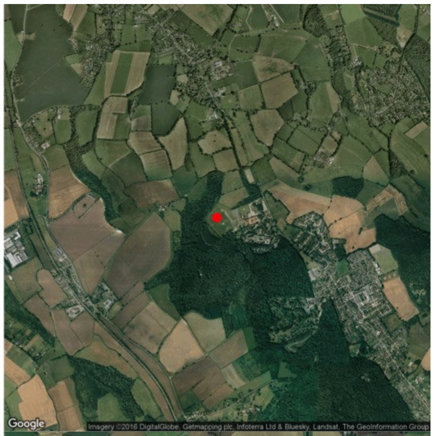
Leeming (17314)	54.2968, -1.53145	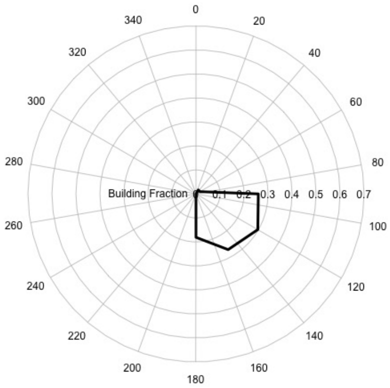	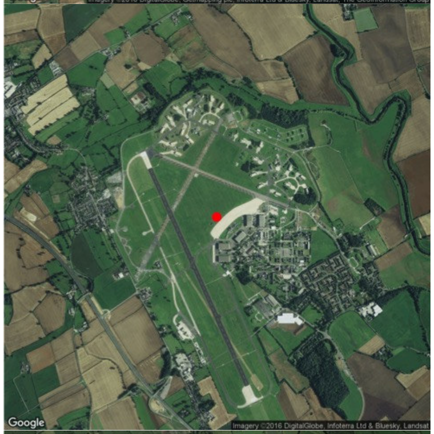
Bristol Lulsgate (18912)	51.3850, -2.71285	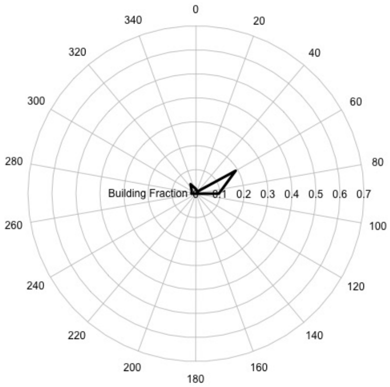	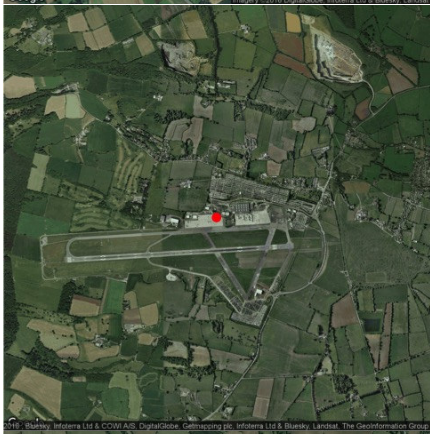
Shepshed (18995)	52.7814, -1.28383	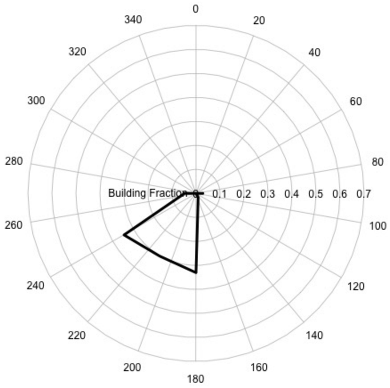	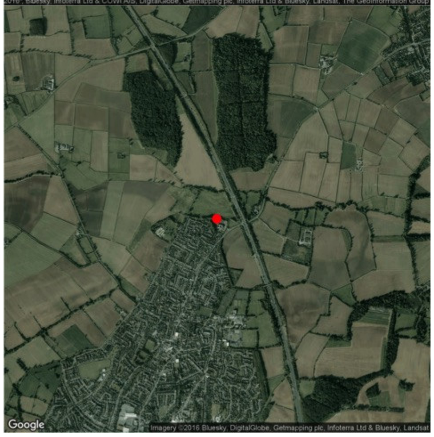

Hereford Credenhill (24996)	52.0796, -2.80102	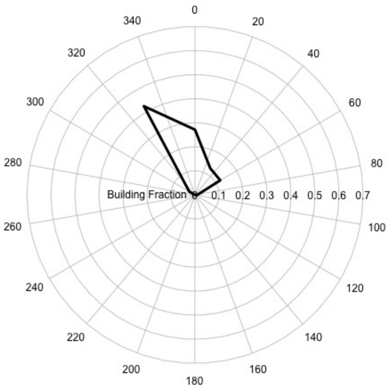	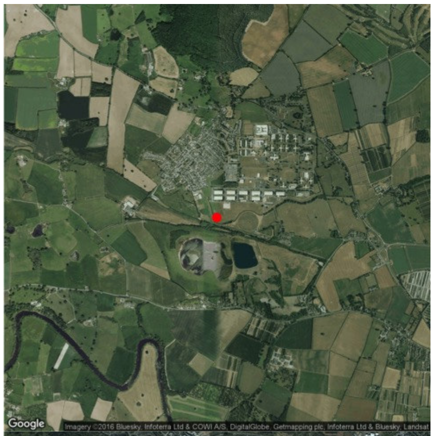
Keele Unviersity Roof (25054)	53.0008, -2.27272	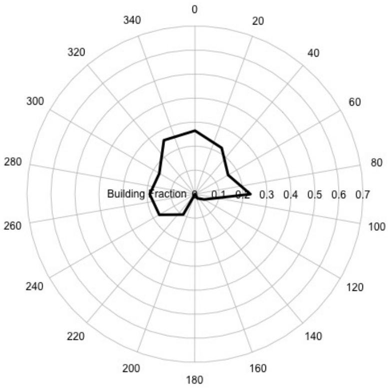	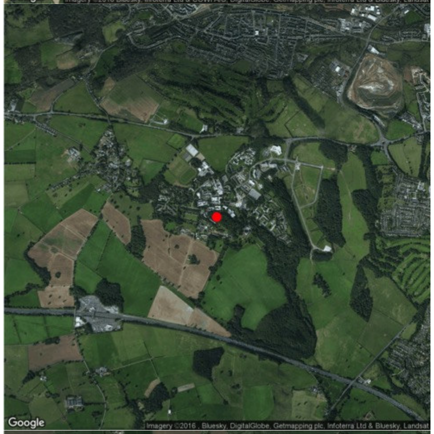
Albemarle (30523)	55.0197, -1.88012	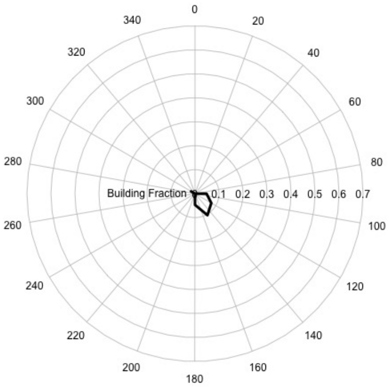	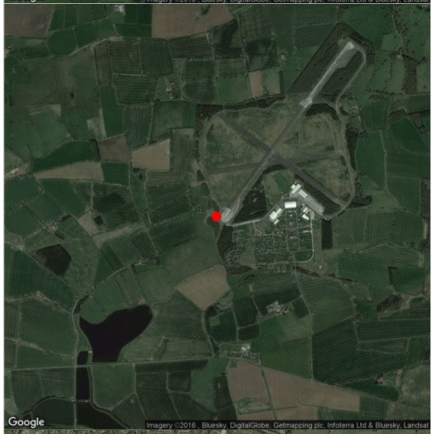
Woodford (55511)	53.3388, -2.15313	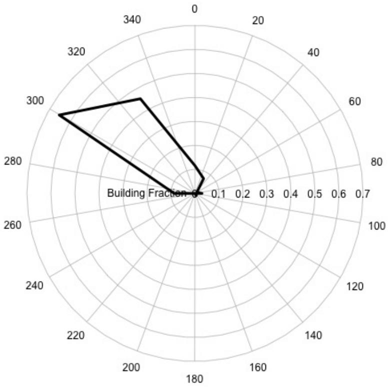	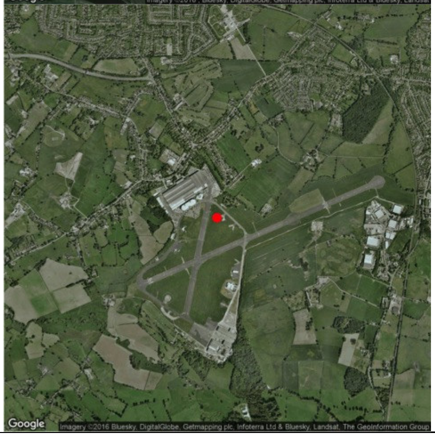
